# Model of a GaAs Quantum Dot in a Direct Band Gap AlGaAs Wurtzite Nanowire

**DOI:** 10.3390/nano13111737

**Published:** 2023-05-25

**Authors:** Daniele Barettin, Igor V. Shtrom, Rodion R. Reznik, George E. Cirlin

**Affiliations:** 1 Department of Electronic Engineering, Università Niccoló Cusano, 00133 Rome, Italy; 2 Faculty of Physics, St. Petersburg State University, Universitetskaya Embankment 13B, 199034 St. Petersburg, Russia; 3 Department of Physics, ITMO University, Kronverkskiy pr. 49, 197101 St. Petersburg, Russia; 4 Department of Physics, Alferov University, Khlopina 8/3, 194021 St. Petersburg, Russia; 5 Institute for Analytical Instrumentation RAS, Rizhsky 26, 190103 St. Petersburg, Russia

**Keywords:** quantum dots, $\vec{k}\cdot \vec{p}$, nanowires, GaAs/AlGaAs, wurtize

## Abstract

We present a study with a numerical model based on $\vec{k}\cdot \vec{p}$, including electromechanical fields, to evaluate the electromechanical and optoelectronic properties of single GaAs quantum dots embedded in direct band gap AlGaAs nanowires. The geometry and the dimensions of the quantum dots, in particular the thickness, are obtained from experimental data measured by our group. We also present a comparison between the experimental and numerically calculated spectra to support the validity of our model.

## 1. Introduction

Polytypism in nanowires represents a unique feature which allows for the engineering of the band structure within a single material [1]. The wurtzite (Wz) phase is not commonly observed at ambient conditions in bulk A${}^{III}$B${}^{V}$ materials, except for nitrides. However, nanowires can be engineered to exhibit the Wz phase, which has generated significant interest in the scientific community due to its potential technological implications [2,3,4,5,6,7]. Al${}_{x}$Ga${}_{1-x}$As nanowires are a promising system for the design of innovative devices. By incorporating the Al component into GaAs [8,9,10], the emission can be tuned over a wide range of wavelengths, allowing for the creation of quantum devices [5,11].

Recent advancements in the accurate control of crystal-phase variation in these structures [12,13] have enabled the growth of strain-free polytypic nanowires along the growth direction [14,15], also at sizes small enough to fabricate quantum dots [16,17,18]. Quantum dots in nanowires are one of the most promising systems for numerous applications in quantum nanophotonics. Recently, several groups have successfully demonstrated nanowire quantum dots in various material systems. However controlled growth of high quality GaAs quantum dots in nanowires remains a challenge. GaAs quantum dots grown in AlGaAs nanowires are expected to exhibit sharper interfaces between the two materials compared to other material systems, as aluminum crystallizes much faster than gallium, leaving the droplet without aluminum once the source is closed [19,20]. This feature allows for better control of the quantum dot shape. Initial studies have already demonstrated the growth of GaAs quantum dots in AlGaAs nanowires. However, the optical properties of these quantum dots were not yet optimal for addressing quantum applications, since the emission line width varied from 95 μeV to 2 meV [21,22]. However, in a more recent study conducted by our group and collaborators [11], we demonstrated the creation of precisely engineered GaAs quantum dots within AlGaAs nanowires, exhibiting a nearly pure crystal structure, high optical quality, and frequency-tuned to the optical transition of Rb atoms. Its growth process involves using the vapor–liquid–solid technique in a molecular beam epitaxy (MBE) reactor on a Si wafer, where a gold particle serves as a catalyst for the growth of the nanowire. To create a quantum dot, the growth of the Al source has to be stopped for a short duration (e.g., 5, 7, or 15 s in the study), leading to the formation of a confined GaAs quantum dot between the AlGaAs segments of the nanowire. This achievement holds significant promise for the development of advanced quantum devices with improved optical properties. Therefore, it becomes essential to use reliable models to study and analyze these structures and to also predict their optoelectronic properties as a function of the growth parameters. Furthermore, based on our current knowledge, there have been no published models in the literature that accurately reproduce experimental results for polytypic materials, such as a GaAs quantum dot in an AlGaAs with Wurtzite crystal symmetry, using reliable numerical parameters. Our group had previously published an innovative study on GaAs quantum dots in an AlGaAs hybrid nanowire structure [23], which consists of alternating zincblende and wurtzite layers. In that publication, we presented a preliminary model with an initial set of numerical parameters. Recently, we have further tested and refined the model by incorporating additional parameters and validating them against experimental data from an AlGaAs nanowire [24]. In this current work, for the first time, we apply this refined model to investigate a GaAs/AlGaAs quantum dot in a wurtzite structure.

Various theoretical models have been employed to study the electronic properties of III–V semiconductors in the Wz phase, including density functional theory calculations based on the GW approximation [25], empirical pseudopotentials calculations with the inclusion of spin-orbit coupling [26], and tight-binding (TB) models [27,28,29]. In particular, Jancu et al. have used a TB model to investigate the electronic structure and optical properties of crystal phase quantum dots (QDs) made by thin zincblende (Zb) layers included in Wz-InP nanowires [27]. Similarly, a tight binding approach has been lately used for the analysis of InP crystal phase QDs [28,29]. Moreover, a GaAs QD embedded in a AlGaAs nanowire has been studied by an 8-band $\vec{k}\cdot \vec{p}$ model [30], which strictly follows experimental data for dimensions, structural, and geometric parameters [23]. In a recent paper a 16-band $\vec{k}\cdot \vec{p}$ model has been implemented to describe the electronic structure of Wz GaAs as well, a paper which also presents a pattern for the determination of the necessary parameters for the calculation of bandstructures in multiband $\vec{k}\cdot \vec{p}$ models [31].

A very relevant study reported the growth of wurtzite AlGaAs nanowires with a wide range of Al content presenting a comparative optical and structural analysis, showing the behavior of the band gap of Wz Al${}_{x}$Ga${}_{1-x}$As, a novelty in the literature [32]. Building upon the experimental findings of the aforementioned study, we have developed a comprehensive model given by an eight-band $\vec{k}\cdot \vec{p}$ model, which incorporates strain and piezoelectric fields, in order to establish the the optoelectronic properties of Wz AlGaAs nanowires with varying Al content [24]. Additionally via our simulations we have calculated in the same paper the band gap of Wz AlAs and the valence band offset between GaAs and AlAs in Wz symmetry by fitting the model to our measurements.

Wz GaAs is believed to have two conduction band minima at the Γ point, which are energetically close to each other: $\Gamma_{7}$ and $\Gamma_{8}$. The energy order of these bands is still under debate [33,34]. In Ref. [35], a complete 10-band $\vec{k}\cdot \vec{p}$ model for wurtzite GaAs was presented. However, in our current work, we have opted to use the well-established 8-band $\vec{k}\cdot \vec{p}$ model from Ref. [24]. We made this choice based on the model’s ability to provide an excellent description of our experimental results.

The paper is organized in the following way: in Section 2, we describe the simulated structures and the applied models. The results of the simulations are shown in Section 3, whereas our conclusions complete the paper.

## 2. Structure and Models

In terms of nanowire morphology, of the structure of the core and of the shell, and of trend of chemical composition, molecular beam epitaxy reactors with a specific Al/Ga ratio yield spontaneously growing nanowires with varying Al percentage in their core and shell. The shell typically has a higher Al content and a higher band gap than the core [32]. Energy dispersive X-ray spectroscopy measurements of the core-shell composition reveal that this is consistently lower than nominal, while the compositional profile of Al in the shell grows nearly linearly with respect to the distance from the catalyst droplet, as a function of the height, ultimately resulting in a conical shell structure [19]. The diameter of the core grows matching the fixed size of the droplet, and transmission electron microscopy investigations have shown that these nanowires display predominantly pure Wz crystalline phase. This process has been similarly detected in InGaAs [36,37], GaAsP [38], InAsSb [39], and GaAsSb [40] nanowires.

Recent investigations have established that realistically simulating the distribution of strain [41] and the quantum confinement [42,43] of a nanostructure is crucial to validate experimental outcomes. To account for the Al concentration variation with height in the shell, we used an algorithm that creates a linear profile of Al content, similar to the experimental composition: the algorithm divides the shell into 100 horizontal layers throughout its height and assigns each layer an Al content that linearly varies from a minimum value at the top of the structure to reach its maximum at the bottom. In this study, we simulated nanowires with an Al proportion of 15% in the core and from 15% to 25% in the shell, in a linear increment from top to bottom [11].

Concerning the QDs, experimentally the growth is achieved by molecular beam epitaxy method using Riber 21 machine on a Si(111) wafer which is firstly outgassed at 850 ${}^{\circ }$C in a separate vacuum chamber before the Au is deposited with total thickness 0.1 nm at 550 °C. After keeping the substrate 1 min at 550 °C in order to improve the homogeneity of the Au droplet size, the substrate was cooled to room temperature and transferred to the growth chamber with no vacuum brake. As the nanowire is grown the substrate temperature is set at 510 °C, the V/III flux ratio is 3 and the total AlGaAs growth rate is fixed at 0.3 nm/s. The AlGaAs is grown for 20 min then the Al source is closed for either 5, 7, or 15 s in order to form the GaAs quantum dot. Afterwards the supply of Al was opened again for 5 min to continue the nanowire growth again to produce a core-shell structure. The growth method results in tapered core-shell nanowires. The tip of the taper is around 20–30 nm while the diameter of the core-shell nanowire is around 150–200 nm. The dimensions of the quantum dot inside the nanowire are accurately controlled by changing the Al shutter closing time (of order of 0.1 s). Due to the very small (<0.14%) lattice mismatch between GaAs and AlGaAs, the positioning of the GaAs quantum dots in the AlGaAs nanowires can be achieved with very high precision. In order to be able to distinguish individual quantum dots, the density of the sample was kept low during growth enabling to distinguish individual nanowires. It appears that for our hybrid system Si wafer is better because quantum dots of a very high optical quality are achieved in comparison with a GaAs wafer, where quantum dots of poor optical quality was obtained.

**Figure 1:**
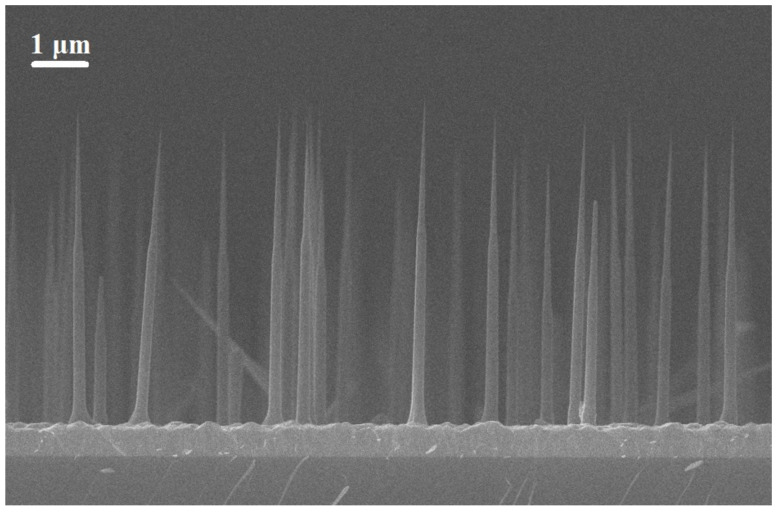
Typical SEM image of AlGaAs NWs with GaAs QDs grown at 510 ${}^{\circ }$C on Si surface.

So in Ref. [11] the growth parameters were chosen to achieve both axial and radial growth, leading to the formation of tapered nanowires. In brief, a spontaneous formation of AlGaAs core-shell NWs in Au-assisted MBE with lower aluminum contents in the cores is a common issue. This feature is explained by a lower diffusivity of aluminum on the NW sidewalls, and hence the vapor–solid growth mode of Al-rich shells. On the other hand, the aluminum content in the cores is determined by the vapor–liquid–solid process in which less aluminum atoms reached the droplet. At the same time, AlAs pairs at the liquid–solid interface crystallize much faster than the GaAs ones. The radial growth process generated a crucial shell that served as a protective barrier for the quantum dot against the external environment. Multiple sets of nanowires were produced with heights between 3–6 μm and core-shell diameters ranging from 150–200 nm at the location of the quantum dot, depending on the growth conditions. As an example, in Figure 1 we present typical SEM image of AlGaAs NWs having nominal Al content 30% with 5s GaAs QDs grown at 51 °C on the Si surface. Geometrically, average NWs height is 5 μm, NWs bottom diameter is ≈250 nm, NWs top diameter is ≈25 nm, and NWs surface density is ≈0.3 NWs/μm${}^{2}$.

The diameter of the quantum dot was determined by the size of the gold catalysts, which was approximately 20 nm. In this article, we have faithfully reproduced these structures: since the growth rate of GaAs ins the MBE is about 0.75 nm per second, the growth times of the dots analyzed in Ref. [11] of 5, 7, and 15 s correspond to a QD height of about 3.75, 4.45, and 11.2 nm, and these are the values we use for our simulations.

The calculations of the band structure for our system were conducted using an 8-band $\vec{k}\cdot \vec{p}$ model [30,44]. In this model, the electron, heavy-hole, light-hole, and spin-orbit split-off bands are described around the Γ point of the Brillouin zone, while all other bands are treated as remote bands. To implement the 8×8 effective-mass Hamiltonian, we followed Foreman’s application of Burt’s exact envelope function theory to planar heterostructures [45,46]. The wave function associated with a state *n* and energy $E_{n}$ is expressed as a linear combination of eight Bloch states, weighted by their respective envelope functions:

(1) $$\begin{array}{c} \psi_{n}\left(r\right)=\sum_{i=1}^{8}\phi_{n}^{i}\left(r\right)u_{i\Gamma }\left(r\right), \end{array}$$

where $\phi_{n}^{i}$ are the envelope functions and $u_{i\Gamma }$ are the Bloch states at *k* = 0 [47].

The electromechanical field within the nanowire and the dot has been computed using a fully coupled continuum model, which has been extensively described in refs. [48,49]. The piezoelectric and strain fields were incorporated into the $\vec{k}\cdot \vec{p}$ method by deformation potentials [50]. The strain parameters of the wurtize crystals were estimated from the corresponding zincblende parameters using the transformations introduced by Martin [51,52], and are consistent with the recent literature [31]. To match the experimental observations detailed in Ref. [32], we utilized free-standing boundary conditions [48]. The piezoelectric features of Wz AlGaAs structures are a topic of debate yet. Bernardini et al. argued that wurtzite InP, GaP, InAs, and GaAs exhibit piezoelectric coefficients that are at least one order of magnitude smaller compared to those of III-N materials [53]. However, several theoretical studies have suggested that these coefficients are still significant and comparable to those of III-N systems, albeit slightly smaller (approximately half) [54,55]. In our work (Ref. [24]), we adopted the piezoelectric coefficients for GaAs from Ref. [56], which were further validated by recent experimental investigations [57]. These coefficients were found to be approximately one-fourth of the average values observed in III-N materials. As for AlAs, the only documented coefficients we are aware of are from Ref. [58], and they are an order of magnitude lower. For a comprehensive discussion and the selection of the electromechanical parameters we adopted, we refer to our recent article [24].

Further, the detailed methodology employed to determine the parameters necessary for the analysis of the band structure, such as the energy gap values of AlAs, GaAs, and AlGaAs alloy in the Wz phase, the bowing parameter, and the valence band offset between AlAs and GaAs, has been thoroughly explained in Ref. [24], as well as the numerical values of these parameters, which for completeness, are provided in Table 1.

In the continuum approach, optical transitions are computed using the dipole matrix elements ${\vec{\mu }}_{nm}$, which are calculated from the momentum matrix element

(2) $$\begin{array}{cc} {\vec{p}}_{nm}\; & \equiv \langle \psi_{n}|\vec{p}\;|\psi_{m}\rangle \\ \; & =\sum_{i,j=1}^{8}\left(\langle \phi_{i}^{\left(n\right)}|\vec{p}\;|\phi_{j}^{\left(m\right)}\rangle \delta_{ij}+\langle \phi_{i}^{\left(n\right)}|\phi_{j}^{\left(m\right)}\rangle \langle u_{i}|\vec{p}\;|u_{j}\rangle \right)\\ & \equiv {{\vec{p}}_{nm}}^{\;(\phi )}+{{\vec{p}}_{nm}}^{\;(u)}, \end{array}$$

where ${\vec{p}}^{,(\phi )}$ and ${\vec{p}}^{,(u)}$ are the envelope and Bloch parts of the momentum matrix element, respectively. Since envelope functions are usually slowly varying at the scale of the primitive cell, they can be neglected [59,60], being much smaller than the Bloch parts. Finally, oscillator strengths are given by [61]:

(3) $$\begin{array}{c} {\vec{P}}_{nm}=\frac{2\pi e^{2}\hbar^{2}}{\epsilon_{0}m_{0}^{2}V}{\left|\hat{e}\cdot {\vec{p}}_{nm}\right|}^{2}, \end{array}$$

where $\hat{e}$ is the unit vector of the electric field direction, *V* is the volume of the structure, *e* is the electron charge, $m_{0}$ is the free electron mass, and $\epsilon_{0}$ is the vacuum permittivity. All models were implemented and solved by using the TiberCAD simulator [62,63].

## 3. Results

Using the models and parameters outlined in the preceding section, we conducted 8-band $\vec{k}\cdot \vec{p}$ calculations that incorporated electromechanical fields. Our aim was to evaluate the quantum confinement in three distinct configurations: an AlGaAs nanowire with an embedded GaAs QD with a height equal to 3.75, 4.45, and 11.2 nm, respectively.

**Figure 2:**
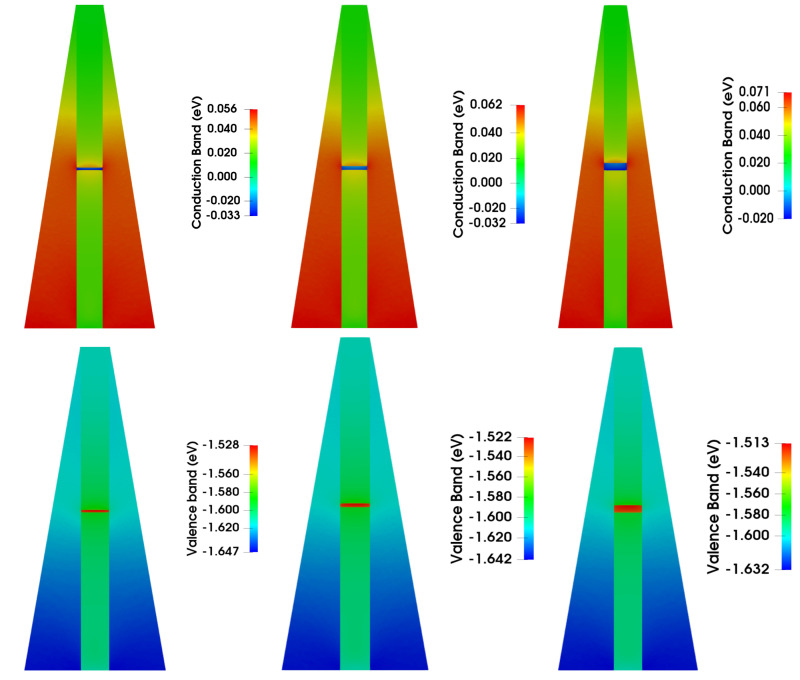
2D plots of conduction (**top**) and valence (**bottom**) bands in three AlGaAs nanowires with an embedded GaAs QD with a height equal to 3.75, 4.45, and 11.2 nm, respectively, from left to right. The core of the nanowire contains 15% of Al, while the shell has an Al content ranging from 15 to 25%, increasing gradually from the top to the bottom (at a temperature of 4 K).

In Figure 2, we present the 2D representation of the conduction and valence bands in an xz plan of these configurations. Regarding the nanowire, it can be observed that the energy gap remains constant in the core region. However, in the shell as the Al concentration increases, the energy gap also increases proportionally. The Al concentration starts from a value comparable to that of the core in the upper part of the structure and increases linearly as a function of distance towards the bottom of the structure, reaching its maximum value.

The minima of the two bands are clearly localized in the QDs, where a quantum confinement is generated in all three dimensions. The slight quantitative variations in the minima are primarily caused by the electrical field created by the piezoelectric and spontaneous polarization [49], aimed within wurtzite symmetrical structures along the growth axis, and recognized as the cause of the quantum-confined Stark effect that confines holes and electrons at the bottom and top of the QD, respectively [64,65,66,67].

**Figure 3:**
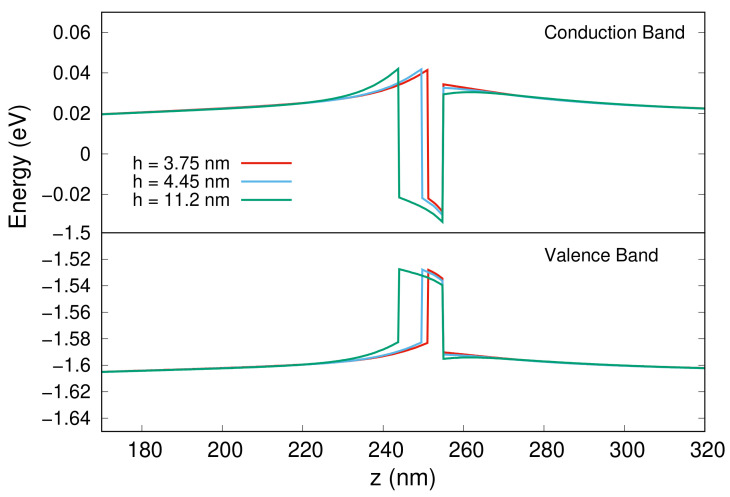
Conduction (**top**) and valence (**bottom**) band structures along the z-axis of the nanowire for the three analyzed structures. The horizontal central black line divides the two different energy scales for electrons and holes, and the energy in the x-axis is referred to the Fermi level.

This appears more evident in Figure 3, where we show a plot of the band structures (the conduction band on top and valence band on bottom) along the vertical z-axis in the center of the nanowire for the three configurations.

Two aspects are clearly observed. First, a tilt of the bands along the growth direction in the region corresponding to the QD due to the electric field. The electrons are thus confined at the top of the QD, while holes at the bottom. This effect, known as we mentioned as quantum-confined Stark effect, appears independent of the height of the dots and reduces the overlap between the wave functions of the charge carriers and generally produces a reduction in the optical emission of wurtzite QDs.

**Figure 4:**
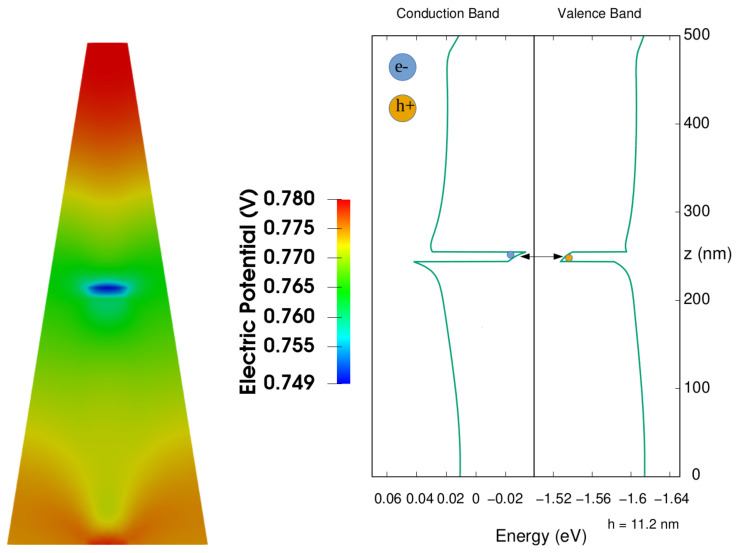
(**Left**): Two-dimensional plot of electric potential (height of QD equal to 11.2 nm). (**Right**): Conduction and Valence band along the growth direction (z) of the nanowire (center of the core). The arrow indicates the transition between holes (h+) and electrons (e−). A vertical central black line divides the two different energy scales for electrons and holes, and the energy in the *x*-axis is referred to the Fermi level.

The second aspect, which instead depends on the height of the QDs, is a variation of the height of the barrier on the top of the QD, quite evident in the case of the conduction band. Both of these characteristics are due to the presence of an electric field oriented along the z axis, as we see in the right part of Figure 4, where, for the case with height of QD equal to 11.2 nm, we show the electric potential in the nanowire, due to the presence of an electric dipole. This is due to the fact that these structures have a wurtzite crystal symmetry given by an alternation of two sublattices, one formed of cations and one of anions. The spatial separation of the sublattices along the vertical z growth-axis results in a spontaneous polarization field oriented in the opposite direction of the growth axis [68,69]. Thus, the structure possess non-negligible built-in electric fields, which adds up to the piezoelectric field generated by the strain, both responsible for the quantum-confined Stark effect which generates the two observed effects.

The shape of the electric potential clearly shows the variation of the electric field along the growth axis, and the presence of a minimum of the potential in correspondence with the dot. For completeness, on the right side of Figure 4, it is shown once more the corresponding valence and conduction bands along the central axis of the nanowire, which can thus be compared with the variations of the electric potential. The arrow in the figure represents the optical transition between the electrons confined in the upper part and the holes in the lower part of the QD.

**Figure 5:**
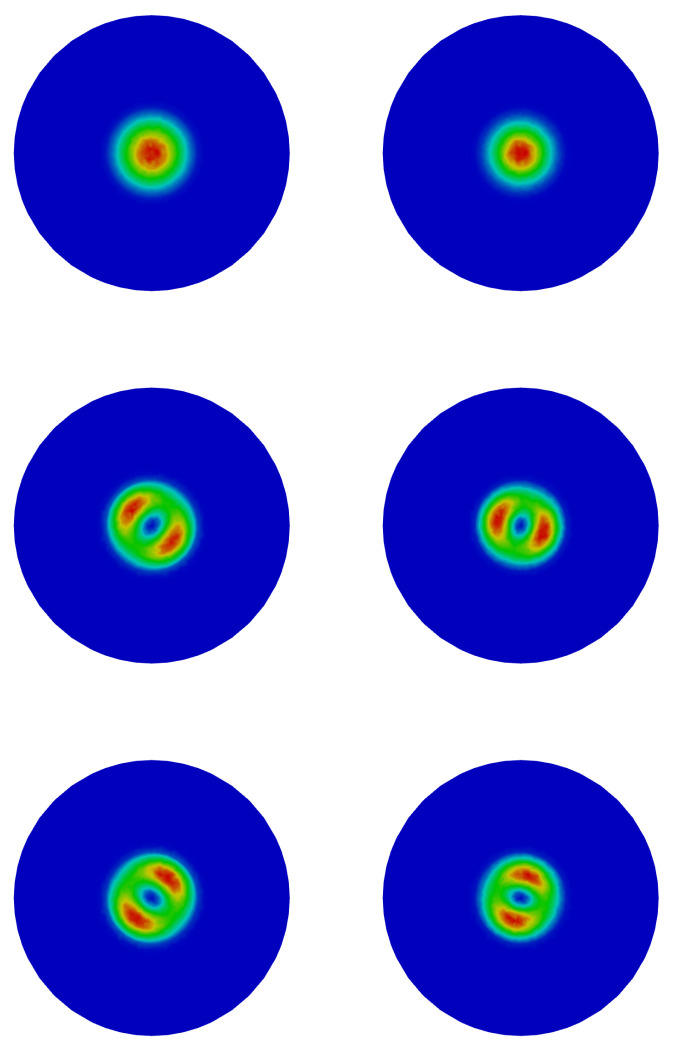
GS (**top**), first (**middle**), and second (**bottom**) excited electron (**left**) and holes (**right**) states.

In Figure 5, we plot in a plane normal to the *z*-axis the probability density for the ground state (GS) and the first two excited states for electrons and holes, again for the case with the height of QD equal to 11.2 nm.

The first and second excited states are energetically degenerate for both electrons and holes. Furthermore, we note that the orientations are practically the same if we compare the two types of carriers. This depends on the fact that, in structures with a wurtzite symmetry, the electric field is completely homogeneous with respect to the radial direction; therefore, there are no favored orientations from an energy point of view on the xy plane, as instead happens in structures with zincblende symmetry [44]. As we have seen, the variations of the electric field occur along the *z*-growth axis, with the consequence of a confinement of the electrons and of the holes, respectively, in the upper and lower part of the QD.

**Figure 6:**
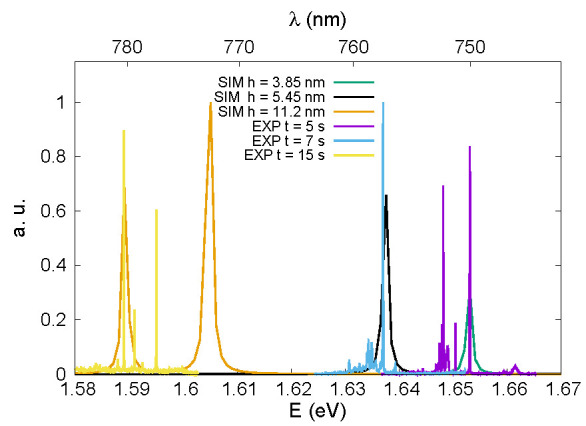
Experimental (EXP) measurements (PL) compared with simulated (SIM) spectra (T = 4 K) for the three analyzed structures.

Ultimately, we juxtapose experimental photoluminescence (PL) spectra analogous to those presented in Ref. [11] against modeled ones in Figure 6, for all three configurations. The optical spectra have been computed assuming complete occupation of all energy states, indicative of high excitation and intensity. Consequently, for a direct comparison with the experimental PL, we scaled both the calculated and measured spectra.

As for the computed spectra, for which we use a single particle model, neglecting the Coulomb interactions, we note that since in the case of the two smaller QDs the quantum confinement is rather limited, we observe the presence of only one peak, corresponding to the GS-GS transition. Instead, in the case of the larger QD, we also have a second peak relating to the transition between the two excited states, which, as we have seen previously, are completely degenerate.

On the other hand, regarding the experimental spectra, the energy levels of the quantum dot are primarily given by the height of the quantum dot, which correspond to growth times in our case. Moreover, the energy levels of quantum dot determine the emission energy observed on photoluminescence (PL) spectra. The low temperature PL spectra of three samples with different growth times 5, 7, and 15 s of the GaAs quantum dot were similarly obtained in previous works (see experimental PL spectra on Figure 5 in Ref. [11]). The optical experiments were performed in a helium cryostat, at a temperature of 4.2 K. The quantum dots were excited above band gap with a continuous-wave laser at 532 nm.

The laser was focused to a spot size of about 1 μm by using a microscope objective (NA = 0.85). The excitation laser light and photoluminescence were both collected through the same objective, (for more details see Ref. [11]). The rich emission spectrum has been attributed to competing charging mechanisms under a non-resonant excitation.

Despite these differences, the simulations and experimental measurements show excellent agreement in terms of the energy position and relative distance of the two peaks on the energy scale. However, since the simulations do not replicate the actual occupation of the electronic states, the absolute intensities of the peaks cannot be compared.

## 4. Conclusions

With an 8-band $\vec{k}\cdot \vec{p}$ model that includes strain and piezoelectric fields by continuum approach, we have simulated the electromechanical and optoelectronic properties of single GaAs quantum dots embedded in direct band gap AlGaAs nanowires. The geometry of the structure, the dimensions of the quantum dots, and the local content of the nanowires have been rigorously derived from experimental data. With the help of our model, we have presented a series of results that thoroughly describe the analyzed structure. Specifically, we have illustrated the quantum confinement through the band structure and the relevance of the quantum-confined Stark effect for a wurtzite symmetry structure, which modifies the mentioned confinement, reduces wavefunction overlap, and can have a drastic impact on the optical properties of a quantum dot. We have also shown the probability density of the charge carriers, which is consistent with previous literature. A final comparison between the experimental and numerically calculated spectra strongly supports the validity of our model.

## Figures and Tables

**Table 1:** Parameters used in the simulations.

Parameters	GaAs Wz	AlAs Wz
a (nm)	0.399	0.4002
c (nm)	0.653	0.654
$\Delta_{cr}\;$ (eV)	0.010	−0.169
$\Delta_{so}\;$ (eV)	0.017	0.019
$m_{e}^{\|}\;$ (eV)	0.202	0.300
$m_{e}^{⊥}\;$ (eV)	0.206	0.320
$A_{1}\;$	−15.22	−7.04
$A_{2}\;$	−2.86	−2.12
$A_{3}\;$	12.36	4.92
$A_{4}\;$	−7.18	−3.46
$A_{5}\;$	−8.79	−4.26
$A_{6}\;$	−12.43	−6.02
$a_{1}\;$ (eV)	−6.8	1.0
$a_{2}\;$ (eV)	−8.6	−7.2
$D_{1}\;$ (eV)	−3.7	−17.1
$D_{2}\;$ (eV)	4.5	7.9
$D_{3}\;$ (eV)	8.2	8.8
$D_{4}\;$ (eV)	−4.1	−3.9
$D_{5}\;$ (eV)	−4.0	−3.4
$D_{6}\;$ (eV)	−5.5	−3.4
$C_{11}\;$ (GPa)	145	141
$C_{12}\;$ (GPa)	51	49
$C_{13}\;$ (GPa)	38	41
$C_{33}\;$ (GPa)	158	149
$C_{44}\;$ (GPa)	38	40
$e_{13}\;$ (C/m${}^{2}$)	0.15	−0.05
$e_{33}\;$ (C/m${}^{2}$)	−0.295	0.04
$E_{g}$ (eV)	1.506	2.232
*C* (eV)	−0.076	Bowing parameter
VBO (AlAs-GaAs Wz) (eV)	−0.368	Valence band offset

## Data Availability

The data presented in the current work are available on request from corresponding authors.

## Abbreviations

The following abbreviations are used in this manuscript:

Wz	Wurtzite
MBE	Molecular Beam Epitaxy
TB	Tight-binding
QDs	Quantum dots
Zb	Zincblende
GS	Ground state
